# Effects of postnatal growth restriction and subsequent catch-up growth on neurodevelopment and glucose homeostasis in rats

**DOI:** 10.1186/s12899-015-0017-5

**Published:** 2015-06-05

**Authors:** Erica E. Alexeev, Bo Lönnerdal, Ian J. Griffin

**Affiliations:** Department of Nutrition, University of California, Davis, CA 95616 USA; Department of Pediatrics, University of California, Davis Medical Center, Sacramento, CA 95817 USA

**Keywords:** Growth restriction, Catch-up growth, Development, Insulin sensitivity

## Abstract

**Background:**

There is increasing evidence that poor growth of preterm infants is a risk factor for poor long-term development, while the effects of early postnatal growth restriction are not well known. We utilized a rat model to examine the consequences of different patterns of postnatal growth and hypothesized that early growth failure leads to impaired development and insulin resistance. Rat pups were separated at birth into normal (N, n = 10) or restricted intake (R, n = 16) litters. At d11, R pups were re-randomized into litters of 6 (R-6), 10 (R-10) or 16 (R-16) pups/dam. N pups remained in litters of 10 pups/dam (N-10). Memory and learning were examined through T-maze test. Insulin sensitivity was measured by i.p. insulin tolerance test and glucose tolerance test.

**Results:**

By d10, N pups weighed 20 % more than R pups (*p* < 0.001). By d15, the R-6 group caught up to the N-10 group in weight, the R-10 group showed partial catch-up growth and the R-16 group showed no catch-up growth. All R groups showed poorer scores in developmental testing when compared with the N-10 group during T-Maze test (*p* < 0.05). Although R-16 were more insulin sensitive than R-6 and R-10, all R groups were more glucose tolerant than N-10.

**Conclusion:**

In rats, differences in postnatal growth restriction leads to changes in development and in insulin sensitivity. These results may contribute to better elucidating the causes of poor developmental outcomes in human preterm infants.

## Background

In term infants, *in utero* growth restriction or small-for-gestational-age status at birth (SGA) are associated with the development of increased adiposity and impaired insulin sensitivity in later life [1], that may be exacerbated by more rapid catch-up growth in the first 1–2 years of life [1, 2]. In comparison, preterm infants grow much more poorly after birth, a term coined *ex utero* growth restriction, and by term corrected age most are below the 5^th^ weight-for-age centile [3]. This *ex utero,* postnatal, growth failure is common in preterm infants, [3, 4] and is associated with poorer neurocognitive outcomes in later life [5, 6]. Further, it has been shown that neonatal leptin deficiency may contribute to adverse neurodevelopmental outcomes associated with postnatal growth restriction [7]. Subsequently, preterm infants have variable amounts of catch-up growth, especially during the first 1–3 years of life [8, 9]. This pattern of small body size at term corrected age, followed by increased rates of growth is similar to that seen in SGA term infants, and there has been concern that this may lead to increased risk of obesity and metabolic disorders arising from impaired glucose tolerance, such as type II diabetes, in preterm infants, similar to the increased risk in term SGA infants [10–12].

We have previously described a rodent model of *ex utero* growth restriction and the effects of variable amounts of catch-up growth on early metabolic and neurocognitive outcomes [13]. Changes in litter size lead to *ex utero* growth restriction (EUGR), and in turn, changes in body composition and poorer neurodevelopment. However, no differences in fasting insulin or glucose in early life were seen [13]. In the present study, we used the same model to assess the effects of *ex utero* growth restriction and subsequent catch-up growth on longer-term metabolic outcomes including glucose tolerance and insulin sensitivity.

The objectives of our study were to examine the effects of early postnatal growth restriction, followed by varying degrees of postnatal catch-up growth on growth (both body size and body composition), insulin sensitivity, glucose tolerance, neurodevelopment, and brain myelination. We hypothesized that early postnatal growth restriction would result in poorer neurodevelopment and lead to improved glucose tolerance and insulin sensitivity. We further hypothesized that in EUGR rats, early catch-up growth would lead to improved neurodevelopment but reduced insulin sensitivity and glucose tolerance compared to EUGR rats that did not have early catch-up growth.

## Results

### Growth

**Fig. 1:**
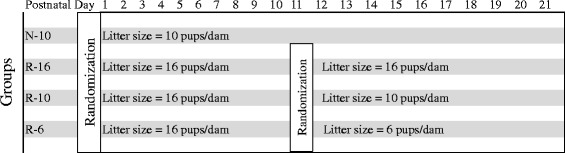
Design of the animal study. On d2, rat pups were randomized to litters of 10/dam (Normal growth (N), five males and five females) or 16/dam (Restricted growth (R), eight males and eight females). On d11, R pups were re-randomized into litters creating catch-up (R-6, 6 pups/dam), normal (R-10, 10 pups/dam) or reduced growth (R-16, 16 pups/dam) groups. N pups remained in litters of 10 pups/dam (N-10)

Growth differed significantly between the normal (N) and restricted (R) intake groups by d5 (14.2 ± 0.19 g vs. 11.4 ± 0.10 g, *p* < 0.001) onwards. By d10 the R groups were approximately 20 % smaller than the N groups (*p* < 0.001, Fig. 1).

On d10, R animals were re-randomized to litters of 6 (R-6), 10 (R-10) or 16 (R-16), while N pups remained in litters of 10 (N-10). The R-16 group remained significantly smaller than the N-10 group throughout the study. The weight of the R-6 pups “caught-up” with the N-10 pups by d15 and were statistically indistinguishable from them for the rest of the study.

**Fig. 2:**
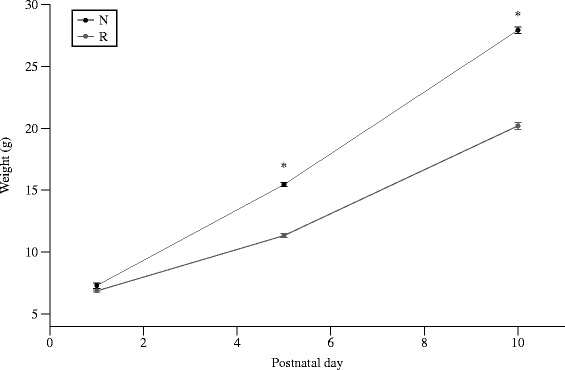
Postnatal weight (g) from d1-10. By d5, R pups were ~ 20 % smaller than N pups. Values are means ± SEM. *Different from N litters, *p* < 0.05

The R-10 group grew intermediate to the N-10 and R-16 animals until d21 (Fig. 2), and was similar to the N-10 and R-6 groups thereafter.

**Fig. 3:**
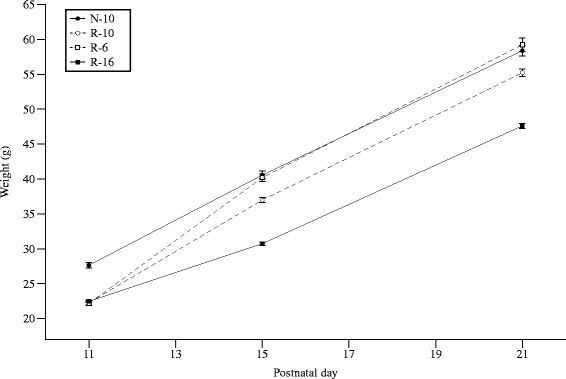
Postnatal weight (g) from d11-21. All R groups diverged by d12. By d15, the N-10 and R-6 groups were similar, the R-16 group showed no catch-up growth, and the R-10 group caught-up half-way between the N-10 and R-16 groups. Error bars represent ± 1 SEM, if not visible they are smaller than the plot symbol

By d40, the R-16 group remained significantly smaller than the three other groups, which were all statistically similar. On d60, the R-16 rats remained significantly smaller than the other three groups. This was seen for both males and females (Fig. 3).

### Body composition

**Fig. 4:**
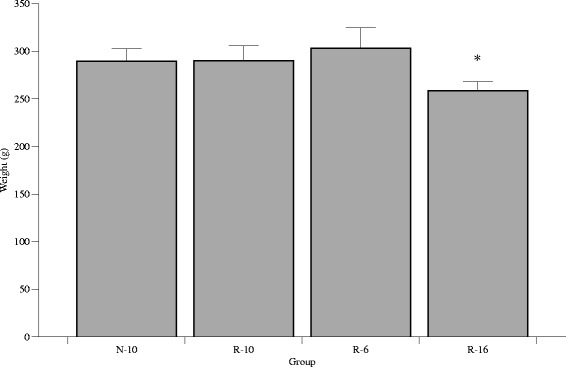
Postnatal weight (g) at d60. The R-16 rats were significantly smaller than the other three groups, and this was seen in males and females. *P < 0.05. Error bars represent ± 1 SEM

Body composition was assessed in a subset of animals (N-10 = 10, R-10 = 10, R-6 = 6, R-16 = 16) at d60. There were no significant differences in percentage water, protein, fat, or ash between the four groups (Fig. 4).

### Serum hormones

On d10, serum leptin was significantly higher in the N group (3.93 ± 0.33 ng/ml) than the R group (1.09 ± 0.31; *p* < 0.0001). Serum triglycerides on d10 were similar in the N (1370 ± 330 mg/L) and R (860 ± 360 mg/L; *p* = 0.77) groups.

Serum leptin on d60 differed significantly between groups, with the R-16 group having the lowest levels (Table 1). Serum triglycerides on d60 were similar among groups, but hepatic triglycerides on d60 differed with the lowest level in the R-10 and R-16 groups and the highest in the R-6 group. Serum insulin values did not differ between groups.

**Table 1:** Fasting glucose, insulin, leptin, and triglycerides in the four groups on d60

Group	N-10	R-10	R-6	R-16	*P* value
Fasting glucose (mg/L)	1084.7 ± 34.4	1057.3 ± 19.3	1080 ± 31.8	1075.2 ± 22.0	NS
Serum insulin (ng/mL)	2.24 ± 0.43	2.41 ± 0.26	2.36 ± 0.61	2.46 ± 0.71	NS
Leptin (ng/mL)	3.74 ± 0.37	4.13 ± 1.12	4.27 ± 0.77	2.79 ± 0.58^a^	P = 0.0037
Serum TG (mg/L)	1603 ± 307	1220 ± 238	1794 ± 487	1450 ± 373	NS
Hepatic TG (mg/L)	1550 ± 263	1100 ± 139^a^	2130 ± 270	1138 ± 194*	P = 0.0203
*n*	20	20	12	32	

Data are expressed as mean ± SEM. *P-*values represent the overall ANOVA p-values. ^a^Denote significant difference from the N-10 group, *p* < 0.05

### Insulin sensitivity

**Fig. 5:**
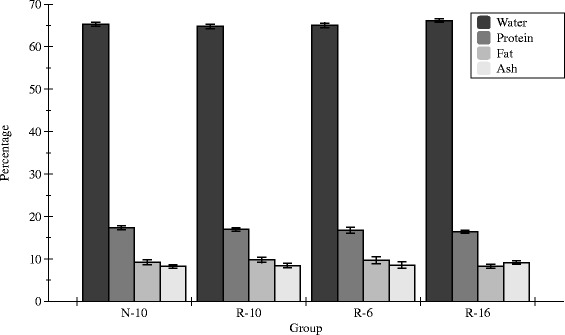
Percentage of water, protein, fat, and ash for each study group at d60. Error bars represent mean ± SEM

Fasting blood glucose on d50 was similar in all four groups (*p* = 0.07). When expressed as the area under the curve (AUC), the two catch-up groups, R-6 (7635 ± 189, n = 23) and R-10 (7531 ± 147, n = 38), had significantly higher AUC than the R-16 group (6870 ± 119, n = 58), while the N-10 group was intermediate between the others (7229 ± 132, n = 47) (Fig. 5). Similar patterns were seen for the AUC between 0 and 30 min and between 30 and 120 min. When individual time-points were considered, the R-6 and R-10 groups had higher blood glucose concentrations than the other groups (N-10 and R-16) at 30, 45 and 60 min.

When the data were examined as change in glucose concentration from baseline, the area under baseline (AUB) between 0 and 30 min was significantly greater for the N-10 group (593 ± 43, n = 47) than for the R-6 group (387 ± 61, n = 58), while the R-10 (428 ± 48, n = 38) and R-16 groups (489 ± 37, n = 58) were intermediate between the two. There were no differences in AUB among the groups for the time period 30 min to 120 min.

### Glucose tolerance

Fasting blood glucose on d55 was significantly affected by sex (M > F; *p* = 0.0061) and by group (*p* = 0.0022). Fasting glucose was lower in the N-10 (99.0 ± 1.4 mg/L) and the R-16 (99.1 ± 1.3 mg/L) groups than in the R-10 group (105.3 ± 1.5 mg/L), with the R-6 group being intermediate between them (102.8 ± 2.0 mg/L).

**Fig. 6:**
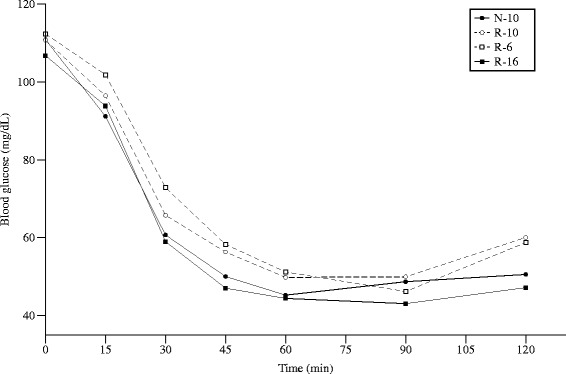
Blood glucose during an intraperitoneal insulin tolerance test (ITT) at d50. Fasting blood glucose was similar in all four groups. Error bars represent ± 1 SEM, if not visible they are smaller than the plot symbol

The AUC was significantly different between groups (*p* = 0.0079) and was greater in males than females (*p* = 0.06). The AUC for the N-10 group (10778 ± 413, n = 47) was significantly greater than both the R-16 (9210 ± 368, n = 59) and the R-10 (8819 ± 453, n = 39) groups, with the R-6 group being intermediate (9620 ± 577, n = 24) (Fig. 6). There were no significant group effects between 0 and 30 min, but were seen subsequently during the remainder of the GTT for the time period 30 to 180 min. A similar pattern was seen in AUB among the groups.

### T-Maze test

**Fig. 7:**
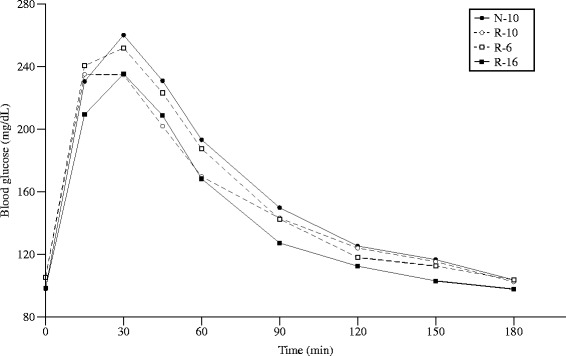
Blood glucose during an intraperitoneal glucose tolerance test (GTT) at d55. Fasting blood glucose was significantly affected by sex (M > F; *p* = 0.0061) and by group (*p* = 0.0022). Error bars represent ± 1 SEM, if not visible they are smaller than the plot symbol

Memory and learning was assessed using spontaneous alternation in a T-maze. The N-10 group scored significantly better (6.86 ± 0.13 successes (n = 69); *p* < 0.05) than any of the other groups (R-6 5.6 ± 0.18 (n = 36), R-10 5.6 ± 0.26 (n = 50), R-16, 5.14 ± 0.15 successes (n = 96)). The effects of group were similar in both sexes (Fig. 7).

### Brain histology

**Fig. 8:**
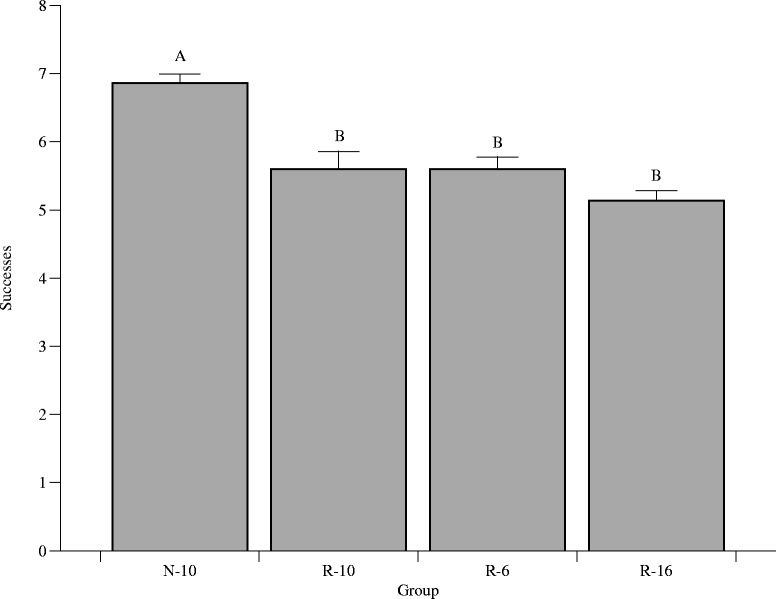
T-maze test success. The N-10 animals scored significantly better (6.86 ± 0.13 successes, *p* < 0.05) than any of the other groups (R-6 5.6 ± 0.18, R-10 5.6 ± 0.26, R-16, 5.14 ± 0.15 successes). Different letters denote significance

The area of MBP-positive fibers in the R-16 group appeared smaller than that in the N-10 group on d60, but no significant differences could be detected. These results suggest that myelination within the hypothalamus and corpus callosum may have been completed by d60 (Fig. 8).

## Discussion

Since poor growth in preterm infants occurs postnatally, we aimed to produce a postnatal model of growth restriction in neonatal rats. Many animal models have been used to examine effects of *in utero* growth restriction with or without catch-up growth on metabolic outcomes, and though these models have provided great insight into infants born small for gestational age or who experience intrauterine growth restriction [14, 15], they do not represent the type of growth that is experienced by most preterm infants. Further, the effects of growth restriction and subsequent catch-up growth on cognition and metabolism have not been examined concurrently. We therefore developed a model of post-natal growth restriction in rat pups based on manipulations in litter size, that we have shown leads to reproducible levels of *ex utero* growth restriction and catch-up growth [13]. This model leads to changes in both milk intake and in growth. However, it is possible that other factors may also be changed by modifications in litter size, for example dam-pup interactions and pup-pup interactions, as seen in other rodents [16, 17].

The initial intervention in our study was carried out from birth until d10 of age, as this period in rats is believed to be equivalent to the third trimester of pregnancy in humans [18], or the period when reduced intake and poor growth are common in premature infants. The increased milk volume intake that occurs as litter size is decreased in the second intervention represents the increased volume intake that preterm infants who experience catch-up growth encounter after hospital discharge. Further, dams of large litters have been shown to produce milk with unaltered protein composition, and thus litter size manipulation results in modified volume intake without altered milk composition [19].

The current study confirms our previous findings that R-6 pups catch-up with N-10 pups by d21, R-10 pups show partial catch-up by d21, and R-16 pups remain smaller than the other three groups [13]. The current study confirms this, but also demonstrates that the R-10 group does ultimately show complete catch-up in body weight by d60. The R-16 group, however, remained significantly smaller than the N-10 group until at least d60.

We have previously shown that catch-up growth in R-6 pups comes at the cost of changes in body composition with R-6 pups having significantly greater percentage body fat, and significantly lower percent lean mass on d21 [13]. The current study demonstrates that by d60, body composition in the R-6 pups has normalized, and is similar to the N-10 pups. Furthermore, although the R-10 pups catch-up to the N-10 pups by d60, the two groups have similar body composition on d60, just as they have at d21. The early changes in body composition related to catch-up group are therefore not maintained over time.

We have previously shown that the R-16 group has lower percentage body fat in d21. By d60, however, the differences in body composition are lost, and all groups have similar percent body fat despite the fact that the R-16 rats remain smaller. Once again, early differences in body composition are not sustained over time. These findings are consistent with the human data, which suggests that although preterm infants with catch-up growth have increased adiposity at term corrected age, those changes are not maintained during the rest of the first year of life [20].

In our previous study there were no differences between groups in fasting insulin or glucose of d21. In the current study we carried out more detailed investigations of glucose homeostasis in older animals. Fasting blood glucose prior to the glucose tolerance test (after a 12 h fast) was significantly greater in the two catch-up groups (R-6 and R-10) than in the groups without catch-up growth (N-10 and R-16). The difference in fasting blood glucose prior to the insulin tolerance test (after a 4 h fast) failed to reach statistical significance. Insulin sensitivity was higher in the groups without catch-up growth (N-10 and R-16) than in the groups that changed their dietary intakes on d10 (R-6 and R-10) and experienced catch-up growth, as shown by their higher AUC values. This occurred even though all groups had similar body composition at the end of the study. It is possible that early changes in body composition may be responsible for the poorer insulin sensitivity seen in the R-6 and R-10 groups in later life, or that changes in early dietary intake or growth lead to long-term changes in insulin sensitivity, possibly via epigenetic mechanisms. Growth restriction may result in improved insulin sensitivity in adulthood since it has been suggested that early undernourishment may enhance insulin sensitivity, as well as fatty acid oxidation [21]. It has been shown that children born prematurely have decreased insulin sensitivity immediately after birth, and those who experience greater weight gain remain having lower insulin sensitivity compared to infants born at term [22].

Conversely, glucose tolerance by GTT was significantly worse in the N-10 group than the R-16 group, as shown by AUC. The two catch-up groups were intermediate between the N-10 and R-16 groups. The differences in fasting blood glucose among the groups is consistent with the findings that mice who are small at birth and have postnatal catch-up growth are at high risk of glucose intolerance [23]; however, there was no significant group effect in AUC for the first 30 min of the GTT, and differences in glucose tolerance were only apparent after 30 min.

Growth before weaning, specifically before d11, could be a critical window for later programming. The developmental origins of disease hypothesis suggests that prenatal development is critical to metabolic adaptation later in life [24]. However, the postnatal environment may be “mismatched” to the early *in utero* environment, creating a disadvantageous phenotype [25]. Cognitive outcomes were worse in the three groups with early growth restriction (R-6, R-10, R-16), and highest in the group with greater early growth (N-10). We thus show that growth restriction, despite catch-up growth, may predispose poor cognition. Though there were no differences in MBP expression at d60, this may be due to the fact that the maximum rate of myelin accumulation in the rat occurs around d20 [26, 27]. Myelin accumulation does continue into adulthood in the rat, though it occurs at a decreasing rate [28]. Several animal studies have shown that dietary restriction during the suckling period results in decreased myelination in early life [29–31]. In our study, early postnatal growth restriction and possible undernutrition due to large litter size may be a cause for the developmental impairments seen in the R groups.

We also examined the effects of growth restriction and catch-up growth on serum hormones, specifically insulin and leptin. Neonatal overfeeding of pups by litter size manipulation has been shown to result in a significant elevation of serum insulin concentration and alterations in hepatic enzymes involved in carbohydrate and lipid metabolism [32]. However, we did not find a significant difference in serum insulin concentrations. Interestingly, serum leptin at d22 and d60 differed significantly between groups, with the R-16 group having the lowest levels. This is consistent with our previous data on d21 [13]. The association of low leptin concentrations in the R litters and poor T-maze score suggests that reduced leptin levels may be a mechanism behind the differences seen in cognition. Leptin has recently been proposed to play a role in brain development during the prenatal and neonatal periods [33]. Administration of leptin to *ob/ob* mice, which are leptin deficient, has been shown to increase brain weight, total brain DNA, and increase MBP-mRNA expression in rodents [34, 35], further suggesting a role for leptin in brain development.

Finally, we demonstrated that hepatic triglyceride content was highest in the group with early catch-up growth (R-6). Hepatic lipid accumulation may be one of the earliest findings in the metabolic syndrome in humans. This, combined with the differences in fasting glucose and in insulin sensitivity, suggests that catch-up growth in this model may be associated with increased risk of metabolic syndrome.

## Conclusion

In summary, we have demonstrated that early growth restriction leads to profound and long-lasting adverse effects on neurodevelopment. Catch-up growth occurs after early postnatal growth restriction, and complete catch-up in weight can occur if it begins before d21 in the rat (equivalent to the first 2–3 years in humans). Postnatal growth restriction without catch-up growth (R-16) leads to short-term reductions in body adiposity, while postnatal growth restriction with catch-up growth (R-6) leads to short-term increases in body adiposity. Neither of these changes in body composition is maintained long-term. Postnatal growth restriction without catch-up growth leads to improved glucose tolerance. However, insulin sensitivity is reduced if catch-up growth occurs after postnatal growth restriction. These finding reinforce the concerns that *ex utero* growth restriction in preterm infants reduces long-term neurocognitive outcomes, and that subsequent catch-up growth may impair insulin sensitivity without improving development.

## Methods

### Animals

**Fig. 9:**
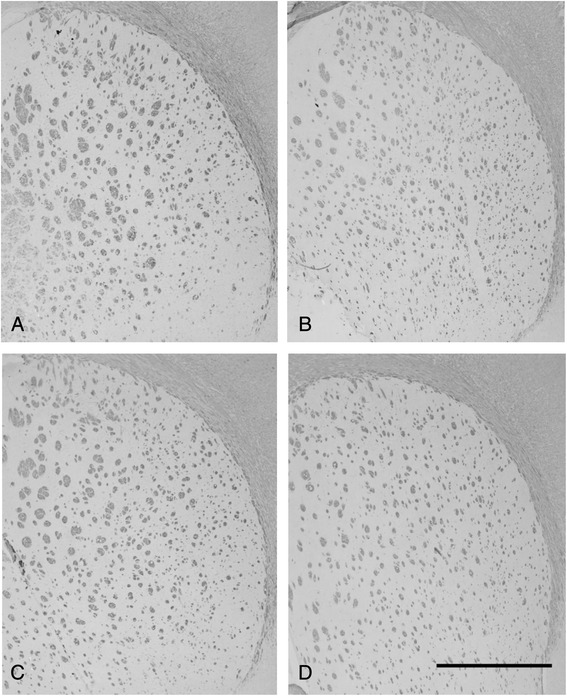
Myelin basic protein (MBP) staining at d60 of (**a**) N-10, (**b**) R-10, (**c**) R-6, and (**d**) R-16 groups. No significant differences in MBP-positive fibers could be detected. Scale bar = 1000 μm

Timed pregnant CD dams were obtained from Charles River (Wilmington, MA) at 14 d of gestation. Rats were housed in solid plastic hanging cages under constant conditions (temperature, 22 °C; humidity, 62 %) with a 12-h dark–light cycle and were allowed to consume food and water *ad libitum*. On d2, rat pups were randomized to litters of 10 pups per dam (Normal growth, N) or 16 pups per dam (Restricted growth, R). On d11, R pups were re-randomized into litters creating catch-up (R-6, 6 pups/dam), normal (R-10, 10 pups/dam) or reduced growth (R-16, 16 pups/dam) groups. N pups remained in litters of 10 pups/dam (N-10). Equal numbers of males and females were included in all litters (Fig. 9). Pups were weaned at d21 to a standard, non-purified rodent diet (LabDiet 5001, Purina, Hayward, CA) fed *ad libitum*. Weights were monitored until d60. The University of California Institutional Animal Care and Use Committee approved all animal procedures.

The period from d2-10 in rats is typically taken to represent the period between the early third trimester and term in humans, and therefore represents early *ex utero* life in preterm infants. The period from d11-21 in the rat is broadly representative of the first 2 years of life in humans, and therefore reflects the period where catch-up growth is common in human preterm infants [18].

### Body composition

A subset of animals had body composition assessed at d60 by carcass analysis. Frozen carcasses were cut and freeze-dried for 24 h to determine water content, calculated from change in weight before and after freeze-drying. Fat content was measured from the change in weight after diethyl ether (Fisher Scientific, Pittsburgh, PA) extraction for 7 d using a Soxhlet apparatus, followed by acetone (Fisher Scientific, Pittsburg, PA) extraction for an additional 7 d. Total ash content was determined following muffle furnace incineration for 72 h at 540 °C and desiccation for 24 h. Protein was calculated as the difference between post-fat extraction weight and ash content. Water, protein, fat and ash content of each animal were expressed as a percentage of total body weight.

### Biochemical analysis

Blood samples were collected at time of sacrifice on d22 and d60. Specimens were centrifuged at 1000 × *g* for 15 min at 4 °C, and serum samples stored at −80 °C until analysis. Serum insulin and serum leptin were measured using ELISA kits (Millipore, Billerica, MA). Serum and hepatic triglycerides were measured with Triglyceride Reagent (Fisher Scientific, Pittsburg, PA) and read at 540 nm at 37 °C.

### Insulin and glucose tolerance tests

An intraperitoneal insulin tolerance test (ITT) was performed on d50 after 4 h of food deprivation. Insulin (0.5 U/kg body weight [36]) was injected intraperitoneally and blood glucose levels were measured in tail vein blood using a glucometer (Easy Plus, Home Aid Diagnostics, Deerfield Beach, FL) at 0, 15, 30, 45, 60, 90, and 120 min after insulin injection. The area under the blood glucose curve (AUC) was calculated using a rhomboid rule. The primary comparison between groups was the total AUC for the entire study (120 min); secondary comparisons were for the AUC between 0 min and 30 min, and between 30 min and 120 min. Larger values for AUC denote poorer insulin sensitivity. In addition, the change in blood glucose from baseline (0 min) was examined. The area under baseline (AUB) was calculated for the entire period, and for the first 30 min and last 90 min separately.

After a 3-days recovery period, an intraperitoneal glucose tolerance test (GTT) was performed after 12 h of food deprivation. Rats were injected intraperitoneally with 2 g/kg of glucose solution (Sigma, St. Louis, MO) and blood glucose was measured at 0, 15, 30, 45, 60, 90, 120, 150, and 180 min after glucose injection [37]. As before, the blood glucose concentrations were used to calculate the area under the blood glucose versus time curve (AUC) for the entire study (0 min to 180 min), as well as for the first 30 min and the last 150 min. Changes in blood glucose from the 0 min baseline were also calculated and the area over the baseline (0 min) value calculated using a rhomboid rule for the time periods 0–180 min, 0–30 min, and 30–180 min.

### T-Maze

Memory and learning were examined by spontaneous alternation in a T-maze on d35. In the T-maze test, rats were tested on their capability to alternate between two directions of an enclosed apparatus in the form of a T placed horizontally, as previously described [38]. Upon successful alternation of direction, animals were given a score of 1. This was repeated ten times, with the maximum score being 9.

### Brain Histology and Immunohistochemistry

For brain histology studies, rats (d60) were deeply anaesthetized with pentobarbital (100 mg/kg) and fixed by transcardial perfusion with 4 % paraformaldehyde. Total brains were removed and placed in 4 % paraformaldehyde solution overnight at 4 °C. Samples were next placed in serial dilutions until fixed in 100 % ethanol and embedded in paraffin. Coronal sections were cut into 8–10 μm sections and immunohistochemically stained with goat polyclonal anti-MBP antibody (sc-13914, Santa Cruz Biotechnology, Santa Cruz, CA) at 1:100 dilution in blocking buffer and donkey anti-goat secondary antibody (sc-2020, Santa Cruz, Biotechnology, Santa Cruz, CA) at 1:500 in 1 % BSA. The staining was developed with DAB substrate (Vector Laboratories, Burlingame, pt]?>CA) and sections were counterstained with toluidine (0.1 %) blue. Images were acquired under microscope at 40X magnification (DP Olympus BX51). Areas of MBP fibers were assessed as MPB-positive per high power field and quantified using ImageJ software (NIH, Bethesda, MD).

### Data analysis

#### Glucose homeostasis

Blood glucose data for the glucose tolerance test are expressed as area under the curve (AUC), calculated using a rhomboid rule. AUC was calculated for the entire study period (AUC_0–180_), for the first 30 min (0–30 min, AUC_0–30_) of the study and for the last 150 min (30–180 min, AUC_30–180_) of the study. Changes in blood glucose from the time-0 baseline are expressed as the area over the time-0 baseline (AOB) for the same time intervals.

Blood glucose data for the insulin tolerance test were converted to AUC, and are expressed for the entire study period (AUC_0–120_), for the first 30 min (AUC_0–30_), and for 30–120 min (AUC_30–120)_. Changes in blood glucose data for the insulin tolerance test are expressed as the area under the baseline (AUB) for the same time intervals.

The primary outcome measure for the glucose tolerance test and for the insulin tolerance tests was the area under the curve (AUC) for the entire study period (AUC_0–120_).

Secondary outcomes for the glucose tolerance test and for the insulin tolerance test was the area under the curve (AUC) for the first 30 min, and for the rest of the study, and the changes in glucose from baseline.

#### Statistical analysis

Weight data were analyzed by repeated-measures ANOVA with age, sex, and group as independent variables.

The effect of group on other continuously distributed outcomes was assessed by ANOVA with sex as a covariant. If main effects ANOVA showed a significant effect of “group”, post-hoc testing to assess differences between the groups was carried out when needed using Tukey’s HSD. All statistical analyses were performed using JMP Pro 11.0 (SAS Institute, Cary, NC) and statistical significance was accepted at *P* < 0.05.

Data are expressed as means ± SEM.
